# A proposed pathway from D-glucose to D-arabinose in eukaryotes

**DOI:** 10.1016/j.jbc.2024.107500

**Published:** 2024-06-27

**Authors:** Elda Iljazi, Rupa Nagar, Sabine Kuettel, Kieron Lucas, Arthur Crossman, Marie-Ange Badet-Denisot, Ronald W. Woodard, Michael A.J. Ferguson

**Affiliations:** 1 Wellcome Centre for Anti-Infectives Research, Division of Biological Chemistry and Drug Discovery, School of Life Sciences, University of Dundee, Dundee, Scotland, UK; 2 D’Arcy Thompson Unit, School of Life Sciences, University of Dundee, Dundee, Scotland, UK; 3 Université Paris-Saclay, CNRS, UPR 2301, Institut de Chimie des Substances Naturelles, Dpt Chemobiologie, Gif-sur-Yvette, France; 4 Department of Chemistry, University of Michigan, Ann Arbor, Michigan, USA

**Keywords:** *Crithidia fasciculata*, glucose metabolism, pentose phosphate pathway, D-arabinose, D-erythroascorbate

## Abstract

In eukaryotes, the D-enantiomer of arabinose (D-Ara) is an intermediate in the biosynthesis of D-erythroascorbate in yeast and fungi and in the biosynthesis of the nucleotide sugar GDP-α-D-arabino*pyranose* (GDP-D-Ara*p*) and complex α-D-Ara*p*–containing surface glycoconjugates in certain trypanosomatid parasites. Whereas the biosynthesis of D-Ara in prokaryotes is well understood, the route from D-glucose (D-Glc) to D-Ara in eukaryotes is unknown. In this paper, we study the conversion of D-Glc to D-Ara in the trypanosomatid *Crithidia fasciculata* using positionally labeled [^13^C]-D-Glc and [^13^C]-D-ribose ([^13^C]-D-Rib) precursors and a novel derivatization and gas chromatography-mass spectrometry procedure applied to a terminal metabolite, lipoarabinogalactan. These data implicate the both arms of pentose phosphate pathway and a likely role for D-ribulose-5-phosphate (D-Ru-5P) isomerization to D-Ara-5P. We tested all *C. fasciculata* putative sugar and polyol phosphate isomerase genes for their ability to complement a D-Ara-5P isomerase-deficient mutant of *Escherichia coli* and found that one, the glutamine fructose-6-phosphate aminotransferase (GFAT) of glucosamine biosynthesis, was able to rescue the *E. coli* mutant. We also found that GFAT genes of other trypanosomatid parasites, and those of yeast and human origin, could complement the *E. coli* mutant. Finally, we demonstrated biochemically that recombinant human GFAT can isomerize D-Ru-5P to D-Ara5P. From these data, we postulate a general eukaryotic pathway from D-Glc to D-Ara and discuss its possible significance. With respect to *C. fasciculata*, we propose that D-Ara is used not only for the synthesis of GDP-D-Ara*p* and complex surface glycoconjugates but also in the synthesis of D-erythroascorbate.

The sugar L-arabinose (L-Ara) is common in plant polysaccharides, such as hemicellulose and pectin, and the conversion of D-Glc to UDP-L-Ara (pyranose and furanose) *via* UDP-D-Glc, UDP-D-glucuronic acid, and UDP-D-xylose is well understood, reviewed in (1). However, the origin of its enantiomer D-arabinose (D-Ara) is not well understood in eukaryotes.

The synthesis and/or uptake of D-Ara by yeast and fungi may be inferred because it is the obligate precursor of erythroascorbic acid, the five-carbon analog of ascorbic acid that is common in these organisms (2, 3).

Until the recent description of Galβ1-4(D-Araβ1-3)GlcNAcβ1-2Manα1-3Manβ1-4GlcNAc in the urine of cancer patients (4), the only other known eukaryotic metabolites of D-Ara were surface glycoconjugates of certain trypanosomatid parasites: the lipophosphoglycans of *Leishmania major* and *Leishmania tropica* (5, 6), the glycoinositolphospholipids of *Endotrypanum schaudinni* (7), and the lipoarabinogalactan (LAG) of *Crithidia fasciculata* (8). In all these cases, D-Ara is present as nonreducing terminal D-arabino*pyranose* (D-Ara*p*) residues and the genes encoding the leishmania D-Ara*p* transferases have been identified (9). Further, the nucleotide sugar donor has been isolated from *C. fasciculata* and structurally characterized as GDP-α-D-Ara*p* (10). The biosynthesis of GDP-α-D-Ara*p* in trypanosomatids is *via* a salvage pathway whereby D-Ara is first activated by an arabinokinase to D-Araα1-phosphate and then condensed with GTP by a pyrophosphorylase to yield GDP-α-D-Ara*p* (10). In *L major*, these two activities reside in the same polypeptide, an arabino/fuco-kinase-pyrophosphorylase that can convert both D-Ara and L-Fuc into GDP-D-Ara and GDP-L-Fuc *via* D-Araα1-phosphate and L-Fuc-1P, respectively (11). Orthologs of the *L. major* arabino/fuco-kinase-pyrophosphorylase genes are also found in other trypanosomatids, including *C. fasciculata*.

Whereas the origin of D-Ara in eukaryotes is unknown, prokaryotes have evolved at least three pathways to make it: (i) The epimerization of polyprenyl-phosphate-D-Rib to polyprenyl-phosphate-D-arabino*furanose* (polyprenyl-phosphate-D-Ara*f*), which acts as donor in the assembly of D-Ara*f*–containing lipoarabinomannan in *Mycobacterium tuberculosis* (12). (ii) The isomerization of D-ribulose to D-Ara by D-arabinose isomerase (13). (iii) The isomerization of D-ribulose-5-phosphate (D-Ru-5P) by D-arabinose-5P isomerase (API) to D-Ara-5P (14, 15), an intermediate in the biosynthesis of the 3-deoxy-D-*manno*-octulosonate component of bacterial lipopolysaccharides. However, there are no obvious homologs of these prokaryotic D-Ara–generating epimerase or isomerase enzymes in the eukaryotes.

Currently, the only data on D-Ara biosynthesis in the eukaryotes are those relating to GDP-α-D-Ara*p* biosynthesis in *C. fasciculata*. Here, it was shown that GDP-α-D-Ara*p* could be labeled efficiently *in vivo* with [2-^3^H]-D-Glc and [6-^3^H]-D-Glc but not with [1-^3^H]-D-Glc (10), suggesting that at least one route to D-Ara might be through the loss of the C1 carbon atom of D-Glc *via* the oxidative branch of the pentose phosphate pathway.

In this paper, we address the route from D-Glc to D-Ara in *C. fasciculata* and other eukaryotes and conclude that in *C. fasciculata*, both arms of the pentose phosphate pathway are involved in converting D-Glc to Ru-5P and that most likely, all eukaryotes can use the isomerase activity of glutamine fructose-6-phosphate aminotransferase (GFAT) to convert D-Ru-5P to D-Ara-5P.

## Results

### D-Glc can be converted to D-Ara *via* the oxidative and nonoxidative arms of the pentose phosphate pathway

**Figure 1:**
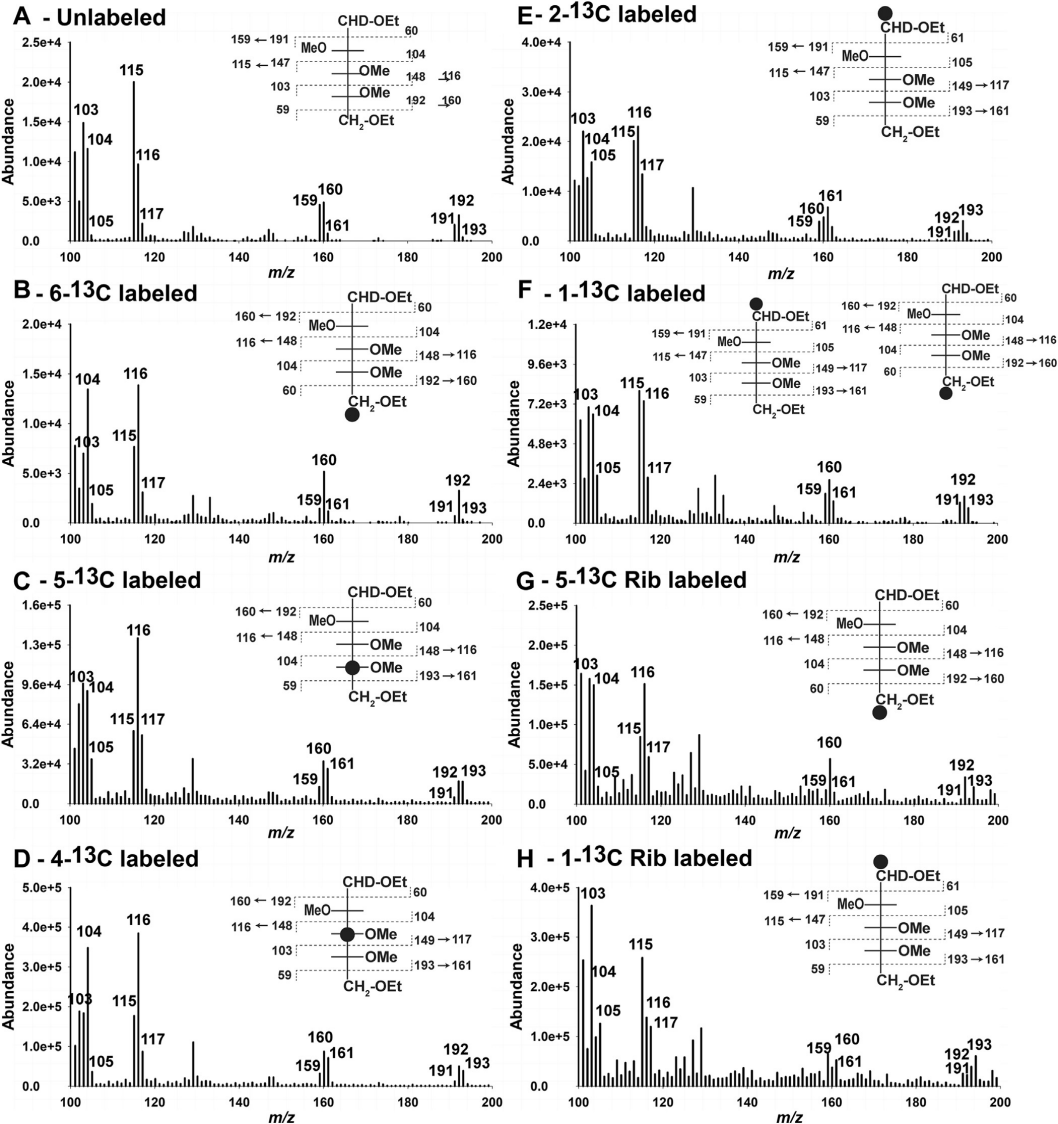
^**13**^**C-labeling of D-Ara in *C. fasciculata* LAG.** A modified methylation linkage analysis procedure (see Experimental procedures) was used to generate partially methylated alditol ethylates (PMAEs) of the constituent monosaccharides of LAG, including the [1-^2^H]-2,3,4-trimethyl-1,5-diethyl-D-arabitol derived from the nonreducing D-Ara*p* residues of LAG, which was isolated and analyzed by GC-MS. The mass spectra of that derivative from LAG purified from *C. fasciculata* grown in unlabeled or positionally ^13^C-labeled Glc or Rib (as indicated in each panel) are shown in panels (*A–H*). The mass spectra shown here are details over the range *m/z* 100 to 200, where most of the key reporter ions are located. The complete spectra are shown in Fig. S1. The position(s) of the ^13^C-atoms in the [1-^2^H]-2,3,4-trimethyl-1,5-diethyl-D-arabitol derivatives inferred by the mass spectra are indicated by *black* dots in the insets of *panels (B–H)*.

*C. fasciculata* cells were grown in a defined glucose-free medium supplemented with D-Glc or with stable isotope labeled D-[6-^13^C]Glc, D-[5-^13^C]Glc, D-[4-^13^C]Glc, D-[2-^13^C]Glc, D-[1-^13^C]Glc, D-[5-^13^C]Rib, or D-[1-^13^C]Rib. The labeled cells were harvested and the major cell surface glycoconjugate, LAG, was extracted, purified, and subjected to a modified gas chromatography-mass spectrometry (GC-MS) methylation linkage analysis procedure (see Experimental procedures). This procedure generates a partially methylated alditol ethylate (PMAE) derivative, [1-^2^H]-2,3,4-trimethyl-1,5-diethyl-D-arabitol, from the nonreducing terminal D-Ara*p* residues of LAG. The electron impact mass spectrum of this derivative, isolated by gas-chromatography, allows us to determine the position(s) of [^13^C] atoms incorporated into the nonreducing terminal D-Ara*p* residues of LAG under the different labeling conditions (Figs. 1 and S1). Inspection of the spectra indicates that the labeling of the terminal D-Ara*p* residues is not complete and, therefore, comparisons with the unlabeled terminal D-Ara*p* PMAE spectrum are needed to aid the interpretations. For example, the *m/z* 59, 103, 115, 159, and 191 ions in the [6-^13^C]-Glc-labeled terminal D-Ara*p* PMAE spectrum (Figs. 1*B* and S1*B*) are reduced in relative abundance compared to those in the unlabeled terminal D-Ara*p* PMAE spectrum (Figs. 1*A* and S1*A*). These ions represent the unlabeled fraction of terminal D-Ara*p* PMAE derivative. At the same time, the increase in relative abundance of the *m/z* 60, 104, 116, 160, and 192 ions in the [6-^13^C]-Glc-labeled terminal D-Ara*p* PMAE spectrum (Figs. 1*B* and S1*B*), compared to those in the unlabeled terminal D-Ara*p* PMAE spectrum (Figs. 1*A* and S1*A*), is consistent with the majority of the [^13^C]-label residing in the five-position of the terminal D-Ara–derived PMAE. The assignments of the principle [^13^C]-label positions are indicated in the insets in (Fig. 1).

The results indicate that the main route from D-Glc to D-Ara involves the loss of the C-1 carbon atom of D-Glc; that is, D-[6-^13^C]Glc, D-[5-^13^C]Glc, D-[4-^13^C]Glc, and D-[2-^13^C]Glc were mostly converted into D-[5-^13^C]Ara, D-[4-^13^C]Ara, D-[3-^13^C]Ara, and D-[1-^13^C]Ara, respectively (Figs. 1, *B*–*E* and S1, *B*–*E*). These data implicate the oxidative branch of the pentose phosphate pathway from D-Glc to D-Ru-5P on route to D-Ara. Consistent with this, when cell labeling was performed with ribose (D-[5-^13^C]Rib and D-[1-^13^C]Rib), the label was retained in the C-5 and C-1 positions of D-Ara (Figs. 1, *G* and *H* and S1, *G* and *H*), also suggesting that D-Ru-5P (the product of ribokinase and Rib-5P isomerase) is on the pathway to D-Ara.

We also found evidence that the nonoxidative transketolase/transaldolase branch of the pentose phosphate pathway also operates in converting D-Glc to D-Ara in *C. fasciculata*, though to a lesser extent than the oxidative branch under the conditions of labeling. Thus, we found that D-[1-^13^C]Glc was converted to a mixture of D-[1-^13^C]Ara and D-[5-^13^C]Ara (Figs. 1*F* and S1*F*). This would be consistent with the conversion of D-Glc to D-Ara *via* D-Rib-5P and/or D-xylulose-5P (D-Xu-5P), which can be readily converted into D-Ru-5P *via* D-Rib-5P isomerase and D-Ru-5P-3-epimerase, respectively.

Taken together, these data lead to the hypothesis that D-Ru-5P is a proximal precursor of D-Ara in *C. fasciculata*.

### GFAT from several species can complement an API mutant of *Escherichia coli*

In the absence of eukaryotic orthologs of the mycobacterial and bacterial enzymes known to lead to D-Ara and taking into account our hypothesis that D-Ru-5P is likely to be a proximal precursor of D-Ara in *C. fasciculata*, we decided to test all identifiable sugar and polyol isomerase genes in the *C. fasciculata* genome (Table 1) for their ability to rescue an *E. coli* API mutant (16).

**Table 1:** *Escherichia coli* API mutant complementation data

Gene Identifier	Species	Description	Codon optimized for *E. coli*	*E. coli* API mutant complementation
BACFN_81317194	*Bacillus fragilis*	API (positive control)	No	Yes (all Figs)
CFAC1_010012400	*Crithidia fasciculata*	Glucose-6-phosphate isomerase CfGPI	No	No (Fig. 2*A*)
CFAC1_190026900	*Crithidia fasciculata*	Phosphomannose Isomerase CfPMI	No	No (Fig. S2*A*)
CFAC1_300033100	*Crithidia fasciculata*	Ribose-5-phosphate isomerase CfRPI	No	No (Fig. S2*B*)
CFAC1_300055100	*Crithidia fasciculata*	Glucosamine-6-phosphate isomerase CfGnPI	No	No (Fig. S2*C*)
CFAC1_210017800	*Crithidia fasciculata*	Triosephosphate Isomerase CfTPI	No	No (Fig. S2*D*)
CFAC1_180028100	*Crithidia fasciculata*	Glucoamine-6-phosphate aminotransferase CfGFAT	No	Yes (Fig. 2*B*)
CFAC1_180028100	*Crithidia fasciculata*	Glucoamine-6-phosphate aminotransferase CfGFAT	Yes	Yes (Fig. S2*E*)
Tb427.07.5560	*Trypanosoma brucei*	Glucoamine-6-phosphate aminotransferase TbGFAT	No	Yes (Fig. 2*C*)
LdBPK_060980.1	*Leishmania donovani*	Glucoamine-6-phosphate aminotransferase LdGFAT	No	Yes (Fig. 2*D*)
LmjF.06.0950	*Leishmania major*	Glucoamine-6-phosphate aminotransferase LmGFAT	No	No (Fig. S2*F*)
LmjF.06.0950	*Leishmania major*	Glucoamine-6-phosphate aminotransferase LmGFAT	Yes	Yes (Fig. S2*G*)
TcCLB.510303.200	*Trypanosoma cruzi*	Glucoamine-6-phosphate aminotransferase TcGFAT	No	No (Fig. S2*H*)
AAH00012.1	*Homo sapiens*	Glucoamine-6-phosphate aminotransferase-2 HsGFAT	Yes	Yes (Fig. 2*E*)
NP_012818.1	*Saccharomyces cerevisiae*	Glucoamine-6-phosphate aminotransferase ScGFAT	Yes	Yes (Fig. 2*F*)

The genome of *E. coli* K-12 contains two genes encoding API activity, KdsD and GutQ (16). Deletion of both KdsD and GutQ produces an auxotrophic strain that requires both D-Ara-5P (to support lipopolysaccharide biosynthesis) and D-Glc-6P (to induce the transport system for D-Ara-5P) to survive and grow (14). Growth of this strain on minimal medium can be rescued by transfection with any API-encoding gene.

**Figure 2:**
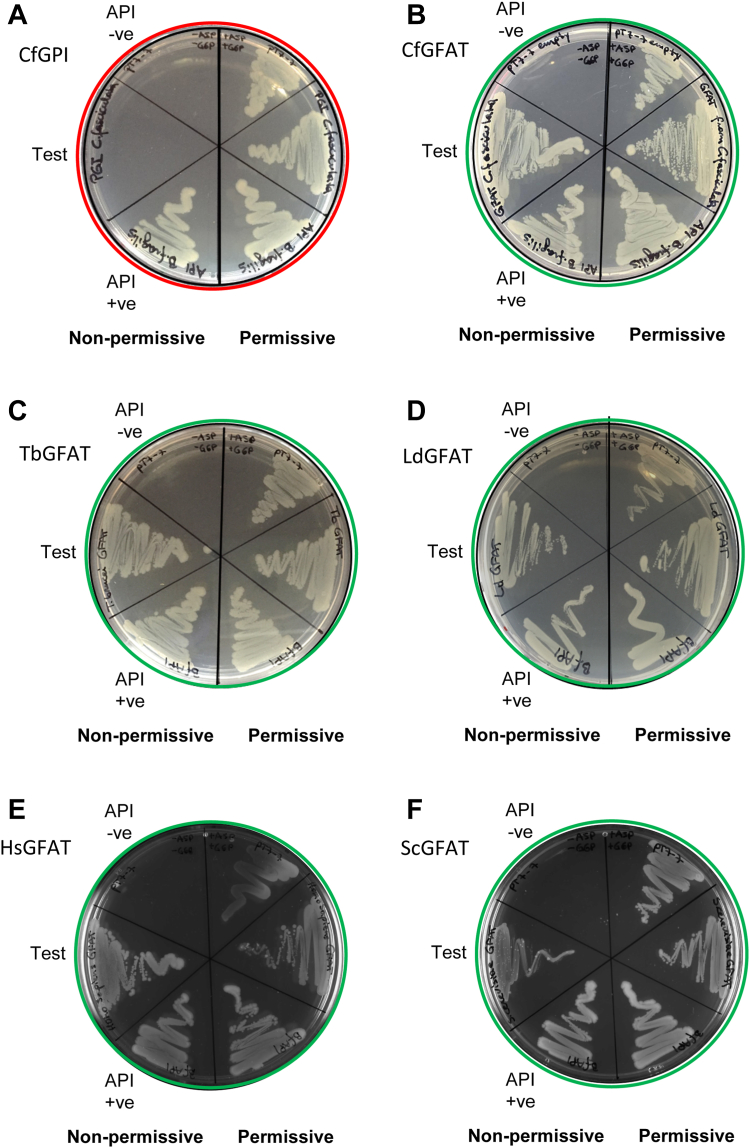
**Eukaryotic GFAT genes can complement an *E. coli* API mutant.** Agar plates containing ampicillin were used to select for bacteria containing the pT7 plasmid. The *right*-hand side (rhs) of the plate was overlaid with D-Ara-5-P and D-Glc-6P which rendered it permissive to growth by the *Escherichia coli* API mutant. The *left*-hand side (lhs) of the plate was nonpermissive to growth by the *E. coli* API mutant. Each side was segregated into three sectors: The top sectors were used to plate the *E. coli* API mutant transformed with an empty pT7 vector. These bacteria should grow on the rhs (permissive) sector, but not the lhs (nonpermissive) sector, acting as a negative control for each experiment. The bottom sectors were used to plate the *E. coli* API mutant transformed with the pT7-API (*Bacteroides fragilis*) vector. These bacteria should grow under permissive (rhs) and nonpermissive (lhs) conditions, acting as a positive control for each experiment. The middle sectors were used to plate the *E. coli* API mutant transformed with one of the pT7-isomerase vectors. All should grow under permissive (rhs) conditions, acting as an additional control for viability, but only under nonpermissive (lhs) if the isomerase gene possesses API activity. The isomerase genes tested are described in (Table 1) and were as follows: *Panel A*: *Crithidia fasciculata* glucose-6-phosphate isomerase (CfGPI). *Panel B*: *C. fasciculata* glucosamine-6-phosphate aminotransferase (CfGFAT). *Panel C*: *Trypanosoma brucei* glucosamine-6-phosphate aminotransferase (TbGFAT). *Panel D*: *Leishmania donovani* glucosamine-6-phosphate aminotransferase (LdGFAT). *Panel E*: *Homo sapiens* glucosamine-6-phosphate aminotransferase-2 (HsGFAT). *Panel F*: *Saccharomyces cerevisiae* glucosamine-6-phosphate aminotransferase (ScGFAT). Experiments where API mutant complementation by indicated the pT7-isomerase plasmid was successful and are ringed in *green*. Experiments where API mutant complementation by indicated the pT7-isomerase plasmid was unsuccessful and are ringed in *red*.

Of the six *C. fasciculata* sugar and polyol isomerase genes we tested (Table 1, Figs. 2, *A* and *B*, and S2, *A*–*D*), only one, GFAT, rescued the *E. coli* API mutant (Fig. 2*B*). To test whether this is a specific trait of *C. fasciculata* GFAT or a more general trait of kinetoplastid GFATs, we transfected the *E. coli* API mutant with additional parasite GFAT genes (from *Trypanosoma brucei*, *Trypanosoma cruzi*, *Leishmania donovani*, and *L. major*). The *T. brucei* and *L. donovani* GFATs complemented the API mutant straight away (Fig. 2, *C* and *D*), whereas the *L. major* and *T. cruzi* GFATs did not (Fig. S2, *F* and *H*). However, when codon-optimized for expression in *E. coli*, the *L. major* GFAT now complemented the API mutant (Fig. S2*G*), as did codon-optimized *C. fasciculata* GFAT (Fig. S2*E*), as expected. Next, we tried codon-optimized human and yeast GFATs and found that both were able to complement the API mutant (Fig. 2, *E* and *F*), suggesting that API activity may be a general feature of eukaryotic GFATs.

From these data, we conclude that many, perhaps all, eukaryotic GFATs are dual function enzymes, making D-glucosamine-6P (D-GlcN-6P) from D-fructose-6P (D-Fru-6P) and glutamine and isomerizing D-Ara-5P and D-Ru-5P.

### Recombinant human GFAT can isomerize D-Ru-5P to D-Ara-5P

**Figure 3:**
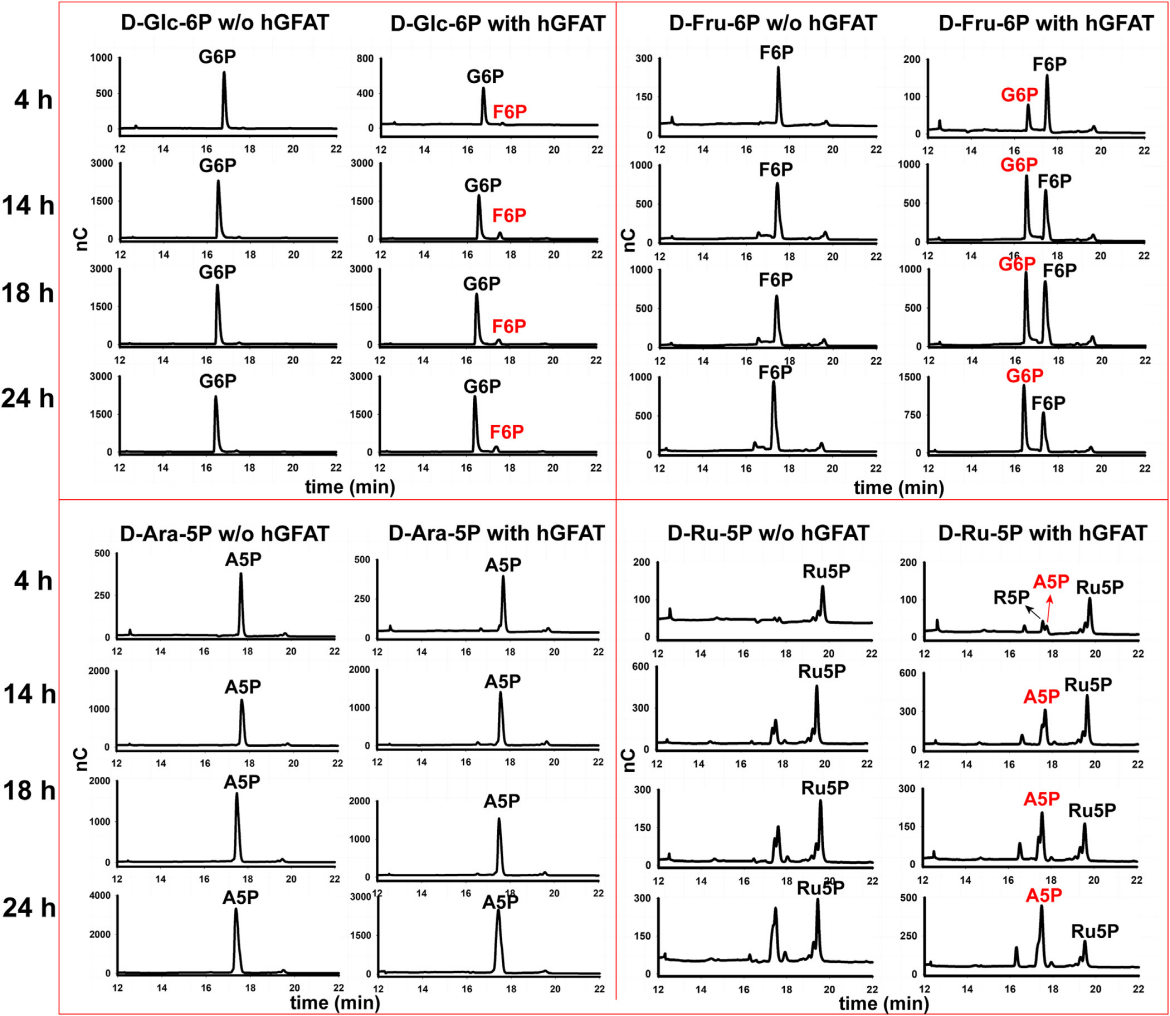
**Human recombinant GFAT can convert D-Ru-5P to D-Ara-5P.** High-pH anion exchange (HPAEC) chromatograms of D-Glc-6P (G6P), D-Fru-6P (F6P), D-Ara-5P (A5P), and D-Ru-5P (Ru5P) incubated without and with recombinant human GFAT (hGFAT) for the times indicated. The detector response (nC) was measured against the retention time. (R5P, D-Rib-5P).

In the absence of glutamine, GFAT will isomerize D-Fru-6P and D-Glc-6P (17). To test our hypothesis from the complementation experiments that eukaryotic GFATs can also isomerize D-Ru-5P to D-Ara-5P, we took purified recombinant human GFAT (Fig. S3) (18) and incubated it with D-Ara-5P and D-Ru-5P and with D-Glc-6P and D-Fru-6P (as controls) and analyzed the products by high-pH anion exchange chromatography (HPAEC) (Fig. 3). The isomerase component of GFAT efficiently converted D-Fru-6P to D-Glc-6P over time (Fig. 3, see F6P with hGFAT panel) whereas isomerization in the reverse direction was limited (Fig. 3, see G6P with hGFAT panel). Thus, the equilibrium in the D-Fru-6P to/from D-Glc-6P isomerization reaction lies to the right, as expected (17). We similarly observed limited conversion of D-Ara-5P to D-Ru-5P (Fig. 3, see A5P with hGFAT panel) but more robust hGFAT- and time-dependent conversion of D-Ru-5P to D-Ara-5P (Fig. 3, see Ru5P with hGFAT panels). The latter was complicated by the spontaneous isomerization of D-Ru-5P to D-Rib-5P and D-Ara-5P under the alkaline conditions of the HPAEC system used to resolve the reactants and products (Fig. 3, see Ru5P without hGFAT panels). Nevertheless, the reduction in D-Ru-5P and concomitant increase in D-Ara-5P compared to D-Rib-5P is apparent in the presence of hGFAT.

### D-Ara is a precursor to D-erythroascorbate in *C. fasciculata*

**Figure 4:**
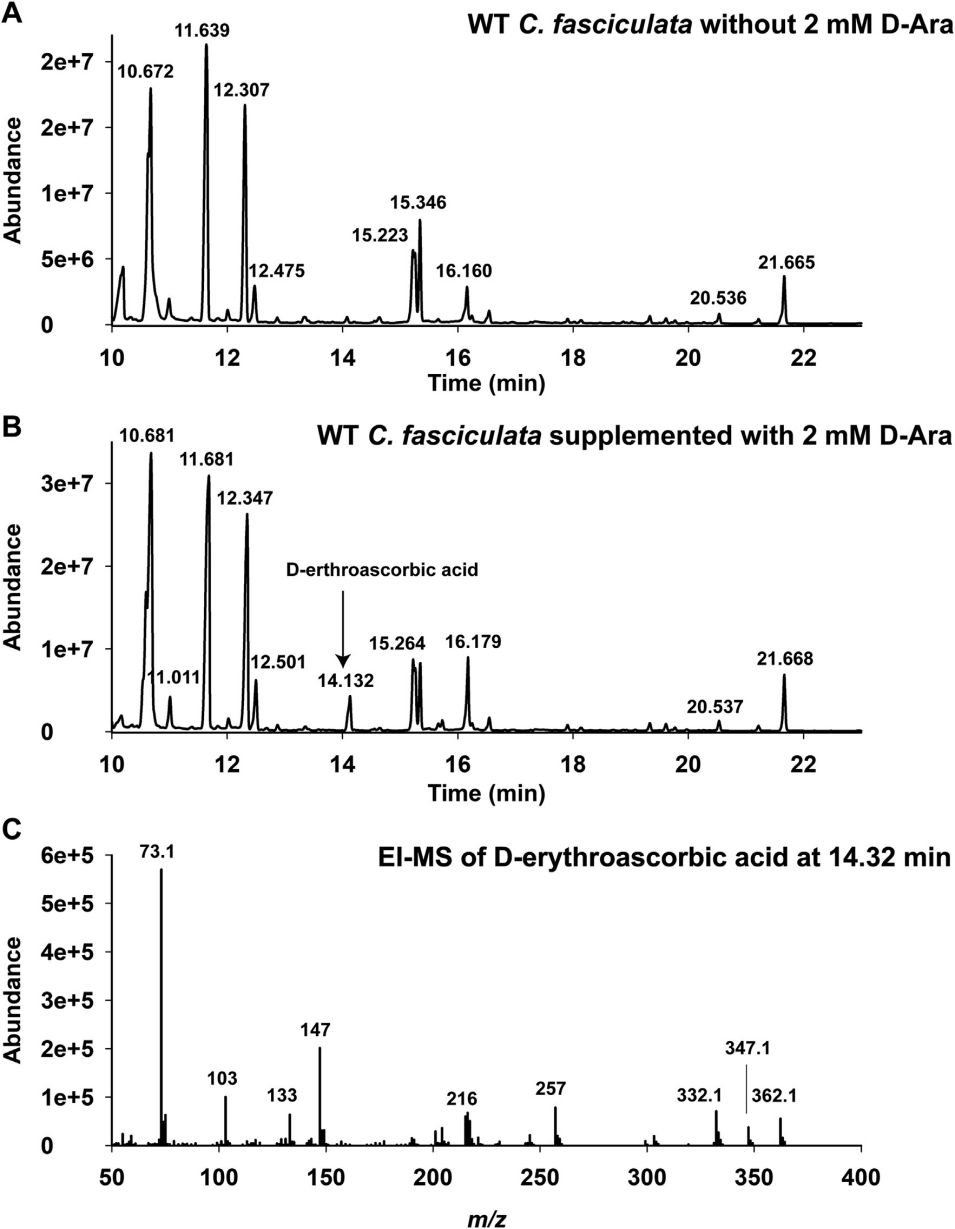
***C. fasciculata* can make D-erythroascorbate from D-Ara.** GC-MS total ion chromatograms of TMS-derivatized polar metabolite extracts of WT *C. fasciculata* grown in the absence (*panel A*) and presence (*panel B*) of 2 mM D-Ara. *Panel C*: The electron impact mass spectrum of the peak labeled D-erythro-ascorbic acid at 14.32 min in *panel B*. According to the National Institute of Standards and Technology (NIST) electron impact spectral database, other peaks in panels A and B are consistent with those of the TMS derivatives of glycyl-glutamate (10.7 min), proline and valine (11.7 min), glutamic acid (12.3 min), phenylalanine (12.5 min), ribose (15.3 min), phosphoglycerate (16.2 min), and *myo*-inositol (21.7 min).

Freshly harvested *C. fasciculata* cells were rapidly chilled to 0 °C to stop metabolism and extracted with solvent to precipitate protein and DNA and to separate polar and apolar metabolites. Aliquots of the polar fraction, corresponding to 1 × 10^10^ cells, were dried and derivatized with trimethylsilyl (TMS) reagent for analysis by GC-MS. A standard of D-erythroascorbate was synthesized, as described in Experimental procedures, and used to determine the retention time and electron impact mass spectrum of the D-erythroascorbate TMS derivative. While no D-erythroascorbate (or D-ascorbate) was found in the polar metabolite extract of *C. fasciculata* grown under normal conditions (Fig. 4*A*), a prominent peak with the same retention time and electron impact mass spectrum as the D-erythroascorbate standard was observed by GC-MS in the polar metabolite extract of *C. fasciculata* grown in the presence of 2 mM D-Ara (Fig. 4, *B* and *C*).

## Discussion

**Figure 5:**
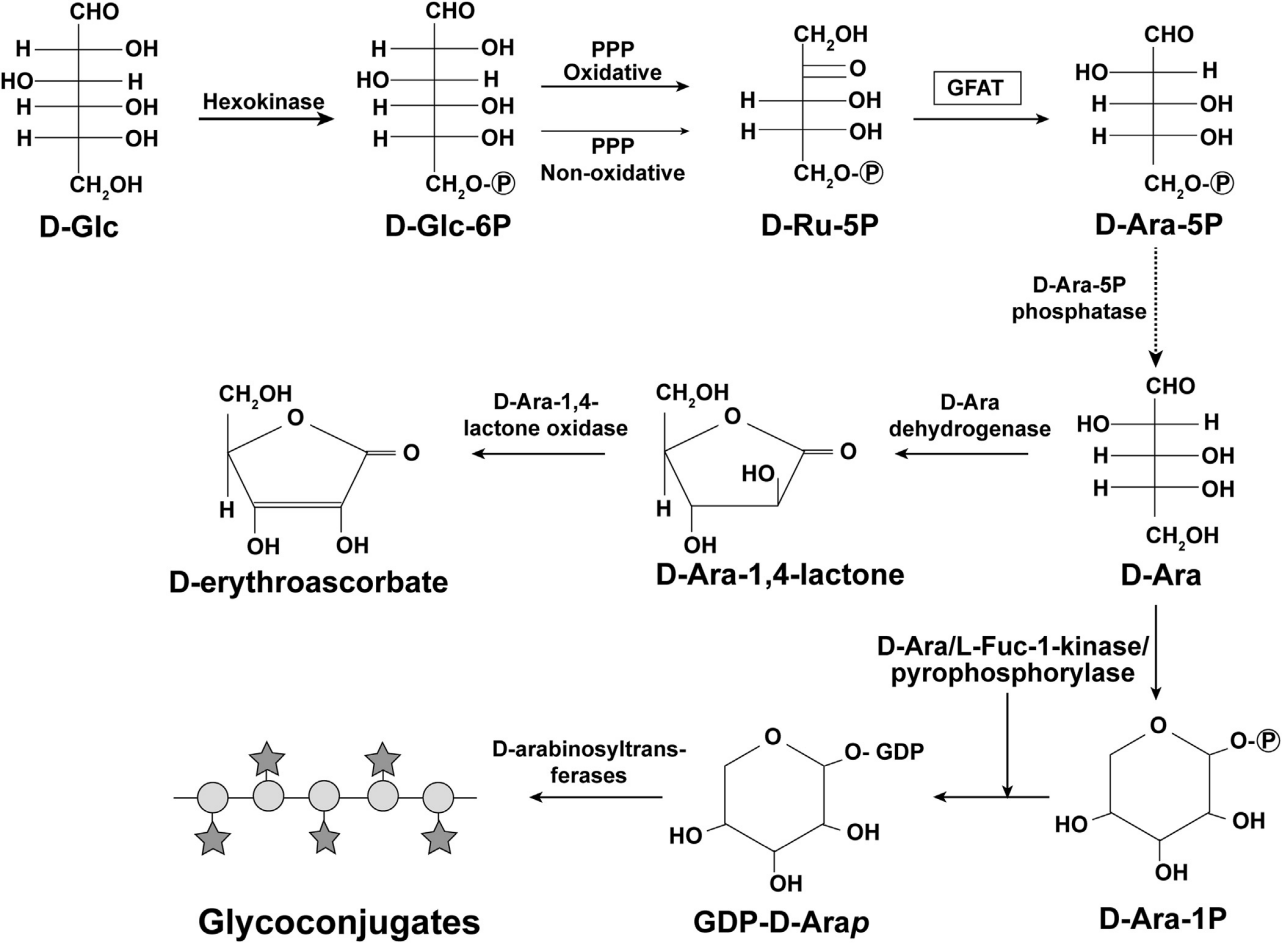
**Proposed pathway for the formation of D-Ara and its metabolites in eukaryotes**. The bioconversion of D-glucose (D-Glc) to D-arabinose is proposed to be *via* D-glucose-6-phosphate (Glc-6P) and both the oxidative and nonoxidative branches of the pentose phosphate pathway (PPP) to D-ribulose-5-phosphate (D-Ru-5P). In *Crithida fasciculata*, the predominant route was the oxidative branch of the PPP. From the work in this paper, D-Ru-5P is proposed to be isomerized to D-arabinose-5-phosphate (D-Ara-5P) by the isomerase domain of glutamine fructose-6-phosphate aminotransferase (GFAT). An unidentified D-Ara-5P phosphatase is postulated (*dotted* arrow) to convert D-Ara-5P to D-Ara. The conversion of D-Ara *via* D-Ara-1,4-lactone to D-erythroascorbate is well described in yeast and fungal metabolism and also appears from this paper to occur in *C. fasciculata.* The conversion of D-Ara to GDP-α-D-Ara*p* has been described in certain kinetoplastid organisms (*C. fasciculata* and *Leishmania major*), as has the incorporation of D-Ara*p* residues from GDP-α-D-Ara*p*, catalyzed by D-arabinosyltransferases, into complex cell surface glycoconjugates of those organisms.

Based on the data presented in this paper, we postulate that D-Ara is made in eukaryotes by biotransformation of D-Glc *via* the nonoxidative and/or oxidative arms of the pentose phosphate pathway to D-Ru-5P, followed by isomerization to D-Ara-5P by GFAT (Fig. 5). We presume that D-Ara-5P is dephosphorylated to D-Ara to then enter the pathway to D-erythroascorbate in yeast and fungi and the pathway to GDP-D-Ara*p* in kinetoplastids (Fig. 5).

The role of D-Ara in the biosynthesis of the antioxidant D-erythroascorbic acid in yeast and fungi is well established, whereby D-Ara is oxidized by NAD(P)^+^-dependent D-Ara dehydrogenases to D-arabino-1,4-lactone, which is further oxidized to D-erythroascorbic acid by D-arabino-1,4-lactone oxidase (2, 19, 20). While enzymes and genes of D-(erythro)ascorbate biosynthesis have been described in trypanosomatids, the actual nature of their endogenous ascorbate antioxidants (*i.e.*, D-ascorbate and/or D-erythroascorbate) has not (21, 22, 23, 24, 25). Thus, the dual function of GFAT described here suggests that trypanosomatids might utilize D-Ara to make D-erythroascorbate antioxidant as well as, in some species, GDP-D-Ara*p* for the biosynthesis of D-Ara*p*–containing cell surface glycoconjugates. We tested this hypothesis by trying to identify ascorbate and/or erythroascorbate in *C. fasciculata* extracts but were unsuccessful unless 2 mM D-Ara was included in the medium, when D-erythroascorbate was readily identified by GC-MS. This result is compatible with *C. fasciculata* using D-Ara for both D-erythroascorbate as well as GDP-D-Ara*p* and LAG synthesis (8, 10). It is also conceivable, based on these data, that trypanosomatids in general, like yeast and fungi, make and utilize D-erythroascorbate as well as or to the exclusion of D-ascorbate.

The authors of a recent report describing D-Ara–containing free N-glycans in the urine of cancer patients (4) speculated that D-Ara may have arisen in these patients from the epimerization of other pentoses, such as ribose, or from bacterial sources in the gut and then entered into nucleotide sugar salvage and glycan fucosylation pathways (D-Ara being a close structural analog of L-Fuc). However, the data here showing ability of human GFAT to produce D-Ara-5P from D-Ru-5P provides an alternative explanation for the origin of D-Ara in human tissue that might be explored.

The dual function of GFAT in making D-GlcN-6P from D-Fru-6P and D-Ara-5P from D-Ru-5P is, in retrospect, not so surprising given that D-Fru-6P and D-Ru-5P are structurally related. However, the possible implications of competition for the GFAT isomerase domain by D-Fru-6P and D-Ru-5P are interesting, with the possibility that D-Ara-5P and/or its downstream metabolites might provide some kind of measure of the balance between flux through the pentose phosphate pathway and the glycolytic/gluconeogenic pathways. The fact that by D-Ara-5P is a potent competitive inhibitor (*Ki* 50 nM) of *T. brucei* phosphoglucose isomerase may be germane here (26). Hopefully, this report may encourage researchers to compare D-Ara-5P and D-Ara levels in mammalian cells and tissues under different conditions.

## Experimental procedures

### Materials

D-[6-^13^C]Glc, D-[5-^13^C]Glc, D-[4-^13^C]Glc, D-[5-^13^C]Rib, and D-[1-^13^C]Rib were obtained from CK Gas Products Ltd. D-[2-^13^C]Glc, D-[1-^13^C]Glc were obtained from Sigma-Aldrich. Pen-Strep (Penicillin, Streptomycin 10′000 U ml^−1^, 10′000 μg ml^−1^) was from Invitrogen. Silica-gel 60 high-performance TLC was from Merck. D-Ara-5P, D-Glc-6P, D-Ru-5P, and D-Fru-6P were obtained from Sigma.

### Cell culture and biosynthetic labeling

*C. fasciculata* strain HS6 was maintained at 27 °C in modified medium (27, 28) and subcultured twice a week with freshly added L-biopterine (50 nM), folic acid (25 nM), and Pen-Strep (10 U or μg ml^-1^) after each passage. The amount of glucose was lowered from the original amount of 1 g to 50 mg per 100 ml media without affecting the growth of *C. fasciculata*. The cells were washed twice with medium containing no glucose before diluting to a concentration of 1.0 × 10^5^ cells ml^−1^ in fresh media containing unlabeled or ^13^C-labeled glucose or ^13^C-labeled ribose (50 mg per 100 ml of medi). Biosynthetic labeling was performed over 48 h until the cells reached a concentration of 2.0 × 10^7^ cells ml^-1^.

For D-ascorbate and D-erythroascorbate analysis, *C. fasciculata* was grown in SDM79 culture medium (29) containing 10% fetal bovine serum and 1× GlutaMAX at 28 °C with and without the addition of 2 mM D-Ara to a cell density of 3 × 10^8^ cells ml^−1^.

### Purification of LAG from *C. fasciculata*

The purification of LAG was based on (8). Cells were grown to mid-log phase (25–100 ml culture; ∼2.0 × 10^7^ cells ml^−1^) and harvested by centrifugation at 4 °C. The cells were washed twice with Tris-buffered saline, pH 7.4. The cell pellet was resuspended with 100 μl water to form a 300 μl cell slurry to which 750 μl methanol and 375 μl chloroform were added (final ratio water: methanol: chloroform 0.8: 2: 1 (v/v/v)). The mixture was vortexed, sonicated for 30 min, and incubated overnight at 4 °C.

After centrifugation, the pellet was extracted a second time with 1.5 ml water: methanol: chloroform 0.8: 2: 1 (v/v/v). The delipidated pellet (containing LAG) was briefly dried under a stream of N_2_ and re-extracted twice with 600 μl 9% butan-1-ol in water, with vortexing and sonication for 15 min. Each time, the suspension was transferred to a 1.5 ml Eppendorf tube and centrifuged at 16,000*g* for 5 min. The combined supernatants were dried and redissolved in 1 ml 0.1 M ammonium acetate containing 5% propan-1-ol (solvent A), filtered through a glass fiber filter, and applied at 8 ml h^−1^ to a 5 ml column of octyl-Sepharose, pre-equilibrated in solvent in A. After allowing the extract to interact with the octyl-Sepharose overnight, the column was washed with 20 ml solvent A at 10 ml h^−1^ and eluted with a 60 ml gradient from solvent A to solvent B (60% propan-1-ol) at 10 ml h^−1^. Fractions of 1 ml were collected and carbohydrate-containing fractions were detected by spotting 1 μl of each fraction on a silica high-performance TLC plate and staining by spraying with orcinol reagent (180 mg orcinol in 5 ml water, mixed with 75 ml ethanol, cooled on ice-water, and mixed slowly with 10 ml c.H_2_SO_4_; stored in the dark at 4 °C) and heating for 5 min with a heat gun. Fractions of interest were dried to remove propan-1-ol and then freeze-dried several times from water to remove the ammonium acetate and stored at −20 °C.

### Modified methylation linkage analysis of LAG

The usual methylation linkage analysis protocol (30) was modified in order to generate PMAEs, instead of partially methylated alditol acetates, in order to better ascertain the position of ^13^C atoms performed in the D-arabitol chain. Samples of purified LAG (approximately 20 μg) biosynthetically labeled with ^13^C-sugars were dried in 2 ml V-bottomed reaction vials (Sigma), dissolved in 50 μl dimethylsulfoxide (DMSO), and mixed with 50 μl of 120 mg/ml ground NaOH suspended in DMSO. After 20 min, an aliquot of 10 μl of methyl iodide was added and incubation continued for another 10 min. Two further aliquots of methyl iodide were added 10 min apart followed by a final 20 min incubation. The permethylation reaction was stopped by the addition of 250 μl dichloromethane and 1 ml 100 mg/ml sodium thiosulphate. After vortexing and separation by gentle centrifugation, the upper aqueous phase was removed, and the lower dichloromethane phase was washed four times with 1 ml water. The washed dichloromethane phase, containing permethylated LAG, was dried under nitrogen and hydrolyzed with 0.5 ml 4M trifluoracetic acid (100 °C, 4 h). The trifluoracetic acid was removed in a Speedvac concentrator (Savant). The resulting partially methylated sugars were reduced with freshly prepared 0.5 M NaB[^2^H]_4_ (3 h at room temperature). Excess 0.5 M NaB[^2^H]_4_ was destroyed with acetic acid and the samples were dried in a Speedvac. Boric acid was removed by drying twice from 0.25 ml 5% acetic acid in methanol and twice from methanol. Residual acetic acid was removed by drying twice from 50 μl of toluene. The resulting partially methylated alditols were dissolved in 50 μl DMSO and mixed with 50 μl of 120 mg/ml ground NaOH suspended in DMSO. After 20 min, an aliquot of 10 μl of ethyl iodide was added and incubation continued for another 10 min. Two further aliquots of ethyl iodide were added 10 min apart followed by a final 20 min incubation. The per-ethylation reaction was stopped by the addition of 250 μl dichloromethane and 1 ml 100 mg/ml sodium thiosulphate. After vortexing and separation by gentle centrifugation, the upper aqueous phase was removed and the lower dichloromethane phase was washed four times with 1 ml water. The washed dichloromethane phase, containing the PMAEs, was gently dried with nitrogen, redissolved in a small volume of dichloromethane, and stored at 4 °C before analysis.

### Cloning and synthesis of *C. fasciculata* and other sugar/polyol phosphate isomerase genes

*C. fasciculata* genes were amplified from *C. fasciculata* genomic DNA by PCR using using high-fidelity Hot Start *Kod* Polymerase (Merck) and primers that introduced 5′-NdeI and 3′-BamHI restriction sites that were used to ligate the PCR products into NdeI and BamHI digested, dephosphorylated, pT7-7 vector. The various pT7-isomerase plasmids were propagated in DH5α *E. coli* and purified by Qiagen minipreps. The GFAT genes from *T. brucei*, *T. cruzi*, and *Leishmania major* and *Leishmania donovani* were similarly amplified from the respective genomic DNAs and cloned into pT7. In some cases, genes codon-optimized for expression in *E. coli* were synthesized (GenScript), amplified by PCR, and ligated into pT7; these included *Saccharomyces cerevisiae* and *Homo sapiens* GFAT genes (Table 1).

### Complementation of an *E. coli* API mutant

The API-deficient D-Ara-5P and D-Glc-6P auxotrophic *E. coli* mutant TCM 15 (ΔkdsDΔgutQ) (16) was cultured in LB medium supplemented with 15 μM D-Ara-5P and 10 μM D-Glc-6P at 37 °C to an A_600_ 0.5 to 0.6. To make them electrocompetent, the culture was chilled in an ice-water bath for 15 min, centrifuged (3635*g*, 20 min, 2 °C), and the cell pellet was resuspended and washed three times in ice-cold 10% glycerol. Aliquots of 25 μl were stored at −80 °C.

Aliquots of 25 μl of electrocompetent *E. coli* TCM15 (ΔkdsDΔgutQ) cells were transferred to cuvettes (2 mm electrode gap, Gene Pulser Cuvette, Bio Rad) containing 0.5 ml of Gene Pulsar electroporation buffer (Bio-Rad) and 1 ng of pT7-isomerase plasmid DNA. A *Bacillus fragilis* API gene in pT7 was used as a positive control for complementation (16). Cuvettes with no plasmid DNA were included as negative controls. The cells were electroporated using Gene Pulser II (BioRad) set at 2.5 kV and 25 μF. After electroporation, the cuvettes were placed for 2 min on ice and the contents transferred to 1.5 ml Eppendorf tubes with 500 μl of LB medium supplemented with 50 mg/ml ampicillin, 15 μM D-Ara-5P and 10 μM D-Glc-6P and incubated for 1 h at 37 °C with shaking. The transformed cells were plated on agar plates (containing LB medium, 50 μg/ml ampicillin, 15 μM D-Ara-5P and 10 μM D-Glc-6P) and left at 37 °C overnight. Single colonies from each plate were inoculated into 5 ml liquid LB medium containing 50 μg/ml ampicillin, 15 μM D-Ara-5P and 10 μM D-Glc-6P and incubated at 37 °C overnight with shaking. Then the cells were washed three times with LB medium, 50 μg/ml ampicillin by centrifugation (3300*g*, 10 min, 4 °C) to remove D-Ara-5P and 10 μM D-Glc-6P. The washed cells were streaked on LB medium, 50 μg/ml ampicillin agar plates with and without D-Ara-5P and 10 μM D-Glc-6P and grown overnight at 37 °C. Only plasmid DNA which complemented *E. coli* TCM15 (ΔkdsDΔgutQ) allowed the mutant to grow under both conditions. Complemented cell colonies growing in the absence of D-Ara-5P and D-Glc-6P were picked, propagated in 5 ml LB medium, 50 μg/ml ampicillin and the pT7 plasmids purified (Qiagen miniprep) for DNA sequencing of their inserts to confirm the identities of the genes conferring complementation of API deficiency.

### Chromatographic analysis of hGFAT activity

The activity of human GFAT (hGAFT) was assayed by incubating 24 μg of purified recombinant hGFAT (18) with 250 nmoles of sugar phosphate substrate (D-Glc-6P, D-Fru-6P, D-Ara-5P or D-Ru-5P) in an assay buffer (20 mM sodium phosphate, pH 7.6, 300 mM NaCl) in a final volume of 25 μl. A parallel no enzyme reaction was carried out for each sugar phosphate substrate as negative control. The reactions were incubated at 37 °C for 4 h, 14 h, 18 h and 24 h after which a 2.5 μl aliquot was diluted in water to a final volume of 40 μl and analyzed using HPAEC chromatography. HPAEC was performed using a Dionex Basic Chromatography System equipped with GS50 gradient pump, ED50 electrochemical detector, and CarboPak PA1 analytical column (2 mm × 250 mm, 10 μm) with the following conditions: 0.0 to 5.0 min isocratic (100 mM NaOH), 6.0 to 30.0 min gradient from 100 mM NaOH to 1 M sodium acetate in 100 mM NaOH, 30.0 to 32.0 min isocratic in 1 M sodium acetate in 100 mM NaOH, 32 to 40 min isocratic in 100 mM NaOH, at a flow rate of 0.25 ml/min.

### D-erythroascorbate analysis by GC-MS

Aliquots of *C. fasciculata* (3 × 10^8^ cells) grown ± 2 mM D-Ara were transferred to centrifuge tubes and chilled for 10 s on dry-ice ethanol to quench cellular metabolism. After centrifugation (1000*g*, 10 min, 0 °C), the cells were washed three times with ice-cold PBS. The final cell pellets were lysed with 500 μl of chloroform/methanol/water 1: 3: 1 (v/v/v) and the lysates were transferred to 1.5 ml Eppendorf microcentrifuge tubes. The Eppendorf tubes were vortexed and incubated for 15 min at 60 °C in a water bath and centrifuged at 16,000*g* for 5 min at 0 °C. The supernatants were transferred to a new 1.5 ml Eppendorf tubes and adjusted with H_2_O to a final ratio of chloroform/methanol/water of 1: 3: 3 (v/v/v). The samples were centrifuged at 16,000*g* for 5 min at 0 °C. The biphasic extract, with precipitated protein and DNA at the interface, contains polar metabolites in the upper methanol/water-rich phase and apolar metabolites in the lower chloroform-rich phase. The clear upper phases were taken and dried in a Speedvac concentrator and redried from 20 μl methanol to ensure the samples were dehydrated. The dried samples were derivatized with 20 μl TMS reagent (dry pyridine, hexamethyldisilazane, trimethylchlorosilane 10: 3: 1 (v/v/v)) for 30 min at room temperature. After incubation, 1 μl aliquots of the TMS-derivatized samples were injected in spitless mode into a GC-MS instrument (HP7890-5975C, Agilent) equipped with a J&W HP-5 ms Ultra Inert GC Column, 30 m, 0.25 mm, 0.25 μm, and using helium as carrier gas (flow: 0.3 ml/min). The injector temperature was 270 °C and the GC gradient was as follows: initial temperature 80 °C, hold 2 min; 30 °C/min to 140 °C; 10 °C/min to 200 °C; 2.5 °C/min to 260 °C; 20 °C/min to 280 °C, hold 10 min.

### Chemical synthesis of D-erythroascorbate

A standard of D-erythroascorbate was prepared according to (31). Briefly, methyl-D-erythro-2-pentulosonate was prepared from commercially available D-ribono-1,4-lactone using phosphoric acid and oxidation with sodium chlorate catalyzed with vanadium pentoxide. The methyl-D-erythro-2-pentulosonate was isolated from the reaction mixture by C18 reverse phase chromatography. The methyl-D-erythro-2-pentulosonate was converted to D-erythroascorbate by refluxing in dry methanol containing sodium acetate in the presence of Amberlite IR-120 (H^+^) ion exchange resin. The D-erythroascorbate product was isolated by C18 reverse phase chromatography. Following TMS derivatization, the D-erythroascorbate-TMS_3_ derivative was found to elute at 14.225 min by GC-MS, under the conditions described above, and its electron impact mass spectrum contained characteristic fragment ions of D-erythroascorbate-TMS_3_ at *m/z* 73, 103, 133, 147, 215, 216, 257, 332 [M^+^-30], and 347 [M^+^-15] along with the [M^+^] molecular ion at *m/z* 362.

## Data availability

All data are contained within the manuscript.

## Supporting information

This article contains supporting information.

## Conflicts of interest

Michael Ferguson reports financial support was provided by Wellcome Trust (Investigator Award 101842/Z13/Z). The authors declare that they have no conflicts of interest with the contents of this article.
