# Supplementary material for: A proposed pathway from D-glucose to D-arabinose in eukaryotes
Source: J Biol Chem. 2024 Jun 27;300(8):107500. doi: 10.1016/j.jbc.2024.107500 (PMC11301363; doi:10.1016/j.jbc.2024.107500)
Supplement: Supporting information [file mmc1.docx]

**A proposed pathway from D-glucose to D-arabinose in eukaryotes.**

Elda Iljazi^1,2^, Rupa Nagar^1^, Sabine Kuettel^1^, Kieron Lucas^1^, Arthur Crossman^3^, Marie-Ange Badet-Denisot^4^, Ronald W. Woodard^5^ and Michael A. J. Ferguson^1,6^

^1^Wellcome Centre for Anti-Infectives Research, Division of Biological Chemistry and Drug Discovery.

^2^Current address: DNA Electronics, [Scale Space White City](https://www.google.com/search?rlz=1C1GCEA_enGB1046GB1046&sxsrf=AB5stBj6vpYANWZhFN-sxTNmMr2IjCj6FQ:1688736552569&q=Scale+Space+White+City&ludocid=10409556237584071980&gsas=1&lsig=AB86z5Xh9WkgZPUANinrCsAjKnca&sa=X&ved=2ahUKEwjz4tby2fz_AhWuWEEAHTHRDWsQ8G0oAHoECEcQAQ), 58 Wood Lane, London W12 7RZ

^3^ D’Arcy Thompson Unit, School of Life Sciences, University of Dundee, Dundee DD1 5EH, Scotland, UK.

^4^ Université Paris-Saclay, CNRS, UPR 2301, Institut de Chimie des Substances Naturelles, Dpt Chemobiologie, 91198 Gif-sur-Yvette, France.

^5^Department of Chemistry, University of Michigan, Ann Arbor, MI 48109-1044, USA.

^6^To whom correspondence should be addressed: e-mail: m.a.j.ferguson@dundee.ac.uk



**Figure S1. ^13^C-labelling of D-Ara in *C. fasciculata* LAG.** Partially methylated alditol ethylates (PMAEs) of the constituent monosaccharides of LAG, were analysed by GC-MS. The mass spectra of derivatives of LAG, purified from *C. fasciculata* grown in unlabelled (Panel A) or positionally ^13^C-labelled Glc or Rib (as indicated in each panel) are shown in panels B-H. The position(s) of the ^13^C-atoms in the [1-^2^H]-2,3,4-trimethyl-1,5-diethyl-D-arabitol derivatives inferred by the mass spectra are indicated by black dots in the insets of panels B-H. Details of these spectra over the range *m/z* 100 – 200 are shown in Figure 1.

**
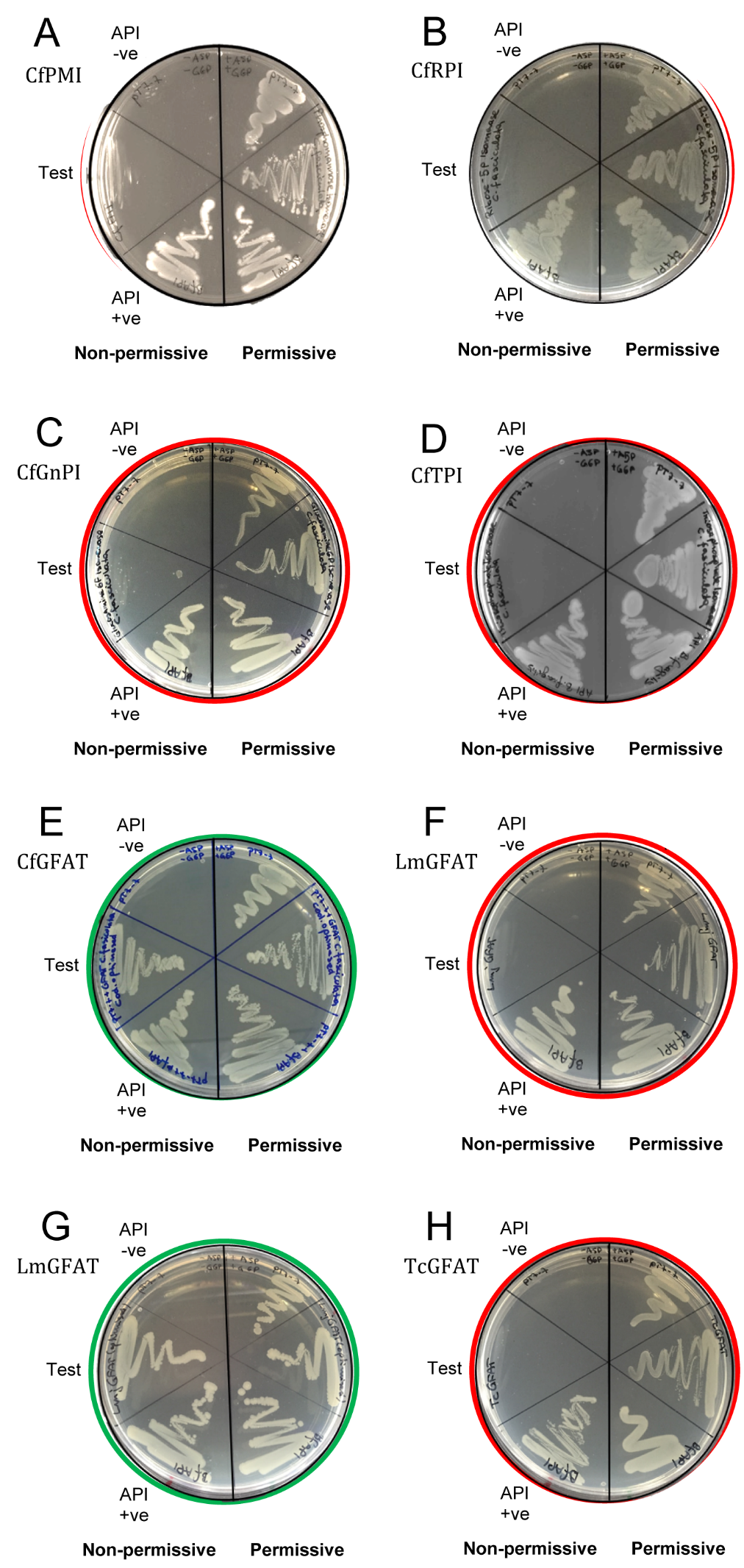
**

**Figure S2. Complementation of an *E. coli* API mutant.**

Agar plates containing ampicillin were used to select for bacteria containing the pT7 plasmid. The right-hand side (rhs) of the plate was overlaid with D-Ara-5-P and D-Glc-6P which rendered it permissive to growth by the *E. coli* API mutant. The left-hand side (lhs) of the plate was non-permissive to growth by the *E. coli* API mutant. Each side was segregated into three sectors: The top sectors were used to plate the *E. coli* API mutant transformed with an empty pT7 vector. These bacteria should grow on the rhs (permissive) sector, but not the lhs (non-permissive) sector, acting as a negative control for each experiment. The bottom sectors were used to plate the *E. coli* API mutant transformed with the pT7-API (*B. fragilis*) vector. These bacteria should grow under permissive (rhs) and non-permissive (lhs) conditions, acting as a positive control for each experiment. The middle sectors were used to plate the *E. coli* API mutant transformed with one of the pT7-isomerase vectors. All should grow under permissive (rhs) conditions, acting as an additional control for viability, but only under non-permissive (lhs) if the isomerase gene possesses API activity. The isomerase genes tested are described in (Table 1) and were: Panel A: *C. fasciculata* phosphomannose isomerase (CfPMI). Panel B: *C. fasciculata* ribose-5-phosphate isomerase (CfRPI). Panel C: *C. fasciculata* glucosamine-6-phosphate isomerase (CGnPI). Panel D: *C. fasciculata* triose phosphate isomerase (CfTPI). Panel E: Codon-optimised *C. fasciculata* glucosamine-6-phosphate aminotransferase (CfGFAT). Panel F: *L. major* glucosamine-6-phosphate aminotransferase (LmGFAT). Panel G: Codon-optimised *L. major* glucosamine-6-phosphate aminotransferase (LmGFAT). Panel H: *T. cruzi* glucosamine-6-phosphate aminotransferase (TcGFAT). Experiments where API mutant complementation by indicated the pT7-isomerase plasmid was successful are ringed in green. Experiments where API mutant complementation by indicated the pT7-isomerase plasmid was unsuccessful are ringed in red.

**Figure S3. Analysis of purified human GFAT used for HPAEC-based activity assays.**

Purified hGFAT was analysed by SDS-PAGE on a 10% gel stained with Coomassie blue. The Precision Plus Protein^TM^ All Blue pre-stained protein standards (BioRad) are at the right.
